# Adrenal Gland and Lung Lesions in Gulf of Mexico Common Bottlenose Dolphins (*Tursiops truncatus*) Found Dead following the *Deepwater Horizon* Oil Spill

**DOI:** 10.1371/journal.pone.0126538

**Published:** 2015-05-20

**Authors:** Stephanie Venn-Watson, Kathleen M. Colegrove, Jenny Litz, Michael Kinsel, Karen Terio, Jeremiah Saliki, Spencer Fire, Ruth Carmichael, Connie Chevis, Wendy Hatchett, Jonathan Pitchford, Mandy Tumlin, Cara Field, Suzanne Smith, Ruth Ewing, Deborah Fauquier, Gretchen Lovewell, Heidi Whitehead, David Rotstein, Wayne McFee, Erin Fougeres, Teri Rowles

**Affiliations:** 1 National Marine Mammal Foundation, San Diego, California, United States of America; 2 University of Illinois, Zoological Pathology Program, Maywood, Illinois, United States of America; 3 National Marine Fisheries Service, Southeast Fisheries Science Center, Miami, Florida, United States of America; 4 Athens Veterinary Diagnostic Laboratory College of Veterinary Medicine, University of Georgia, Athens, Georgia, United States of America; 5 NOAA National Ocean Service, Marine Biotoxins Program, Charleston, South Carolina, United States of America; 6 Florida Institute of Technology Department of Biological Sciences, Melbourne, Florida, United States of America; 7 Dauphin Island Sea Lab and University of South Alabama, Dauphin Island, Alabama, United States of America; 8 Institute for Marine Mammal Studies, Gulfport, Mississippi, United States of America; 9 Louisiana Department of Wildlife and Fisheries, Baton Rouge, Louisiana, United States of America; 10 Audubon Aquarium of the Americas, New Orleans, Louisiana, United States of America; 11 National Marine Fisheries Service, Office of Protected Resources, Silver Spring, Maryland, United States of America; 12 Mote Marine Laboratory, Sarasota, Florida, United States of America; 13 Texas Marine Mammal Stranding Network, Galveston, Texas, United States of America; 14 Marine Mammal Pathology Services, Olney, Maryland, United States of America; 15 National Centers for Coastal Ocean Science, National Ocean Service, Charleston, South Carolina, United States of America; 16 National Marine Fisheries Service, Southeast Regional Office, St. Petersburg, Florida, United States of America; University of Missouri, UNITED STATES

## Abstract

A northern Gulf of Mexico (GoM) cetacean unusual mortality event (UME) involving primarily bottlenose dolphins (*Tursiops truncatus*) in Louisiana, Mississippi, and Alabama began in February 2010 and continued into 2014. Overlapping in time and space with this UME was the *Deepwater Horizon* (DWH) oil spill, which was proposed as a contributing cause of adrenal disease, lung disease, and poor health in live dolphins examined during 2011 in Barataria Bay, Louisiana. To assess potential contributing factors and causes of deaths for stranded UME dolphins from June 2010 through December 2012, lung and adrenal gland tissues were histologically evaluated from 46 fresh dead non-perinatal carcasses that stranded in Louisiana (including 22 from Barataria Bay), Mississippi, and Alabama. UME dolphins were tested for evidence of biotoxicosis, morbillivirus infection, and brucellosis. Results were compared to up to 106 fresh dead stranded dolphins from outside the UME area or prior to the DWH spill. UME dolphins were more likely to have primary bacterial pneumonia (22% compared to 2% in non-UME dolphins, *P* = .003) and thin adrenal cortices (33% compared to 7% in non-UME dolphins, *P* = .003). In 70% of UME dolphins with primary bacterial pneumonia, the condition either caused or contributed significantly to death. Brucellosis and morbillivirus infections were detected in 7% and 11% of UME dolphins, respectively, and biotoxin levels were low or below the detection limit, indicating that these were not primary causes of the current UME. The rare, life-threatening, and chronic adrenal gland and lung diseases identified in stranded UME dolphins are consistent with exposure to petroleum compounds as seen in other mammals. Exposure of dolphins to elevated petroleum compounds present in coastal GoM waters during and after the DWH oil spill is proposed as a cause of adrenal and lung disease and as a contributor to increased dolphin deaths.

## Introduction

A large, multi-year cetacean unusual mortality event (UME) has been ongoing in the northern Gulf of Mexico (GoM) since February 2010, continuing into 2014 [1]. This event has involved predominantly (87%) common bottlenose dolphins (*Tursiops truncatus*) (hereafter referred to as ‘dolphins’) stranded in Louisiana, Mississippi, and Alabama [2]. The UME coincided with the *Deepwater Horizon* (DWH) oil spill, the largest marine-based spill in U.S. history [3]. During and following the DWH oil spill, significantly elevated polycyclic aromatic hydrocarbon (PAH) levels attributed to this spill were detected in coastal GoM waters, including Louisiana, Mississippi, and Alabama [4]. These locations coincided with the states most impacted by the ongoing UME since the DWH oil spill [2]. Dolphin strandings, however, were elevated during March and April before the spill, necessitating an investigative approach including numerous potential causes [1,2,5]. Combined oil exposure, an unusually cold winter during 2011, and fresh water infusions have been proposed as potential causes contributing to this UME [6].

Barataria Bay, Louisiana was one of the heaviest oiled coastal areas from the DWH oil spill, including visualized oiling from the spill encompassing 40 km and 366,000 m^2^ of Barataria Bay’s shoreline lasting in decreasing amounts for at least 2 years [7–10]. The presence of increased coastal PAH levels associated with the DWH oil spill, especially near Grand Isle, Louisiana in Barataria Bay have been confirmed [4]. Further, within the time period of January 2010 to June 2013, the longest lasting cluster of dolphin strandings throughout the northern GoM was in Barataria Bay (August 2010 through 2011) [2]. During the DWH oil spill and response period, numerous dolphins, including dolphins in Barataria Bay, were observed swimming through visibly oiled waters and feeding in areas of surface, subsurface, and sediment oiling [11].

Due to the extensive oiling in Barataria Bay, health assessments were conducted on live dolphins in this area during the summer of 2011 [11]. Barataria Bay dolphins had a high prevalence of moderate to severe lung disease and blood value changes indicative of hypoadrenocorticism; specific blood changes included low serum cortisol, aldosterone, and glucose, and high neutrophil counts [11]. Nearly half (48%) of Barataria Bay dolphins were given a guarded to grave prognosis for long-term survival [11]. The DWH oil spill was proposed as a contributor to adrenal gland and lung disease in live Barataria Bay dolphins.

Previous to the ongoing event, there have been ten dolphin GoM UMEs since 1991, as well as one large die-off during 1990 that occurred before the UME declaration process [1, 12–15]. The majority (82%) of previous dolphin GoM events had brevetoxicosis or morbillivirus as confirmed or suspected causes [1]. While brevetoxicosis events do not leave a histologic signature in affected dolphins, brevetoxicosis-related events are often associated with known algal blooms and deaths that appear to be acute in otherwise healthy-looking dolphins [15]. In prior events classified as brevetoxicosis-related, 50% or more sampled dolphins were positive for brevetoxin with most at high concentrations [15]. Similarly, past UMEs that have been attributed to morbillivirus involved successful detection of morbillivirus in greater than 60% of dolphins tested. [13,16]. There is evidence that *Brucella*, which is commonly found in marine mammals worldwide, can cause disease in cetaceans, including bottlenose dolphins [17–21]. As such, there was a need to evaluate all of these potentially important diseases as playing contributing or leading roles in the ongoing UME.

To assess contributing factors and causes of deaths for stranded UME dolphins following the DWH oil spill, tissues were histologically evaluated from 46 carcasses that stranded in Louisiana, Mississippi, and Alabama, including 22 from Barataria Bay, from June 2010 through December 2012. Perinatal dolphins, stranded dolphins that were less than 115 cm in body length that likely died during late-term pregnancy or shortly after birth, were excluded from this study. On the basis of the live dolphin health assessment findings from Barataria Bay, this study included a focused evaluation of adrenal, lung, and liver lesions with the expectation that if stranded dolphins had been impacted by the DWH oil, they would have lesions consistent with the clinical evidence indicating lung disease and hypoadrenocorticism found in live dolphins. Other potential causes of and contributors to dolphin deaths were investigated, including the presence of histologic lesions and diagnostic test results consistent with brevetoxicosis, morbillivirus infections, and brucellosis. Results were compared to a reference group of fresh dead dolphins from North Carolina, South Carolina, Texas, and the Gulf coast of Florida that stranded prior to or remote from the UME and DWH oil spill timeframes and geographic location.

## Materials and Methods

Tissues and data from bottlenose dolphins that stranded dead or stranded and died were used in this study in coordination with NOAA’s Marine Mammal Health and Stranding Response Program (MMHSRP, T. Rowles) and NOAA’s Southeastern United States Marine Mammal Stranding Network. Only samples from stranded dolphins were included in this study. No animal was killed for the purposes of this study.

### UME dolphins

This study includes common bottlenose dolphins (*Tursiops truncatus*) with a total body length greater than or equal to 115 cm that stranded in Louisiana, Mississippi, or Alabama from June 2010 through December 2012. Dolphins with a total body length less than 115 cm were likely either near-term pregnancy losses or died soon after birth. Carcasses included in this study were either characterized as stranding fresh dead by the responding stranding network personnel or stranded alive then died, had archived tissues available for histologic evaluations, and had tissues not compromised by decomposition based on histologic evaluations by a pathologist. Fresh dead dolphins were the focus of this study to enable the most reliable histologic evaluations which could be compared to other diagnostic test results. Blood-based clinical pathology data were not available from dead, stranded dolphins as a direct comparison to the indices of hypoadrenocorticism detected in the previously published, live dolphin health assessment.

### Reference dolphins

A total of 106 fresh dead reference dolphins, each with a total body length greater than or equal to 115 cm and an existing histopathology report, were compared to UME dolphins in this study [22]. Of these, 51 (48%) reference dolphins had archived tissues available for standardized assessment by a common pathologist (K. Colegrove) heretofore called the ‘standardly scored reference subset’. Reference dolphins for this study stranded in North Carolina and South Carolina from 1996 through 2012, or from Texas or the Gulf coast of Florida from 2002 through 2009 (Table 1). Histologic lesion comparisons from reference dolphins were made to the UME dolphins using both the full reference group and the standardly scored reference subset group. It is possible that some of the reference dolphins may have been part of historical UMEs, specifically brevetoxin UMEs in Florida. Potential brevetoxicosis reference dolphins were not excluded from this study to ensure there was no bias against this as a cause of the ongoing UME.

**Table 1:** Total number, stranding dates, and locations of 152 freshly dead stranded non-perinatal (greater than or equal to 115 cm in total body length) common bottlenose dolphins (*Tursiops truncatus*) from the ongoing northern Gulf of Mexico cetacean Unusual Mortality Event (UME) and reference groups.

Study group	Number	Observed stranding dates	State or Parish
UME dolphins	46	JUN 2010—DEC 2012	AL (n = 6), LA (n = 28), MS (n = 12)
Barataria Bay, Louisiana UME dolphin subset	22	SEP 2010—OCT 2012	Jefferson (n = 16), Plaquemines (n = 3), Lafourche (n = 3)
Reference dolphins	106	APR 1996—AUG 2012	FL (n = 49), NC (n = 2), SC (n = 47), TX (n = 8)
References dolphins—standardly scored subset	51	FEB 2002—JUL 2009	FL (n = 35), NC (n = 2), SC (n = 6), TX (n = 8)

Standardly scored subset references had both histopathology reports and archived tissues available for evaluation.

### Sample collection and processing for histologic examination

Post mortem examinations of UME and reference dolphins were conducted by members of NOAA’s Southeastern United States Marine Mammal Stranding Network either in the field or at institutional facilities. Stranding demographic, temporal and spatial data were collected using a standardized Level-A data record (NOAA Form 89–864 (rev.2007) OMB No. 0648–0178) according to approved NOAA protocols at the time of data collection. Level A data used for this study were the most recently available when extracted from the NOAA’s MMHSRP database during September 2014.

Tissue samples from most major organs of stranded dolphins were collected in 10% neutral buffered formalin, many of which included adrenal glands, bladder, brain, eye, heart, kidney, liver, lung, lymph node(s), muscle, pancreas, reproductive organs, skin, small and large intestines, spinal cord, spleen, thymus, tongue, and trachea. Tissues were processed routinely, embedded in paraffin, sectioned at 5 μm, and stained with either hematoxylin and eosin (HE) (UME dolphins, South Carolina reference dolphins, and Gulf coast of Florida reference dolphins) or hematoxylin, phloxine, and saffron (HPS) (Texas reference dolphins). While this study focused on lung, adrenal gland, liver, lymph nodes and brain to test our hypothesis, full sets of tissues from the UME cases, including the above organs, were evaluated by a pathologist and used for the cause of death determination. Among the 10 UME dolphins with primary bacterial pneumonia, gram stains were completed on all dolphins, and Ziehl Nielson acid fast stains were completed on lung sections of four dolphins. Not all tissues for all dolphins were available for both histologic and other diagnostic tests (e.g. there may have been formalin-fixed lung for histologic evaluation, but no frozen lung for morbillivirus molecular diagnostics). As such, the total numbers of tissues varied throughout the study based upon their availability for each investigation.

### Histologic grading

Targeted evaluation of lesions in select organs (adrenal gland, brain, lung, lymph node, spleen, and liver) from UME dolphins was performed by a board certified veterinary pathologist (K. Colegrove) through evaluation of HE or HPS stained slides. To standardize comparisons among tissues, the same evaluation was completed on the reference dolphins by the same pathologist (K. Colegrove) through evaluation of stained slides (n = 51, standardly scored reference set) or thorough interpretation of lesion descriptions with or without images from histopathologic reports (n = 55) written by other pathologists. To reduce bias, severity grades were carried out blindly without knowledge of the animal identification number.

#### Adrenal gland

The adrenal gland was evaluated quantitatively by measurement of corticomedullary ratios (thickness of cortex divided by thickness of medulla). Corticomedullary ratios were determined similarly to previously published methods on mid-sagittal or mid-cross sections of adrenal glands from 36 UME and 44 reference dolphins using an Olympus BX-41 microscope and Olympus DP-BSW Version 03.02 measuring software [23]. Corticomedullary ratios in dolphins remain constant along the length of the adrenal gland, indicating that ratios calculated on mid- sagittal or mid-cross sections are adequately representative of the entire adrenal gland [24]. Three corticomedullary ratios were obtained from three different representative sites along the section and averaged. For most UME and reference dolphins, only one appropriate mid-sagittal or mid-cross section of either right or left adrenal gland was available for evaluation. Adrenal corticomedullary ratios from 44 reference dolphins were used to statistically define ‘thin’ and ‘thick’ adrenal gland cortices. Thin was defined as a corticomedullary ratio less than the 10^th^ quantile among reference dolphins (0.447), and thick was a corticomedullary ratio greater than the reference’s 90^th^ quantile (0.838). UME and reference dolphins were excluded from evaluation of corticomedullary ratios when mid-sagittal sections of adrenal glands were not present or sections had been cut in a way that precluded precise measuring of cortex or medulla thickness. Corticomedullary ratios were obtained from both the left and right adrenal glands of ten reference dolphins, and using a Fisher’s exact test, measurements were comparable between the left and right sides (*P* = 0.85). Presence or absence of vacuolation of corticocytes in the zona fasciculata was also evaluated.

#### Lung

Lung inflammation severity was graded as mild, moderate, or severe based on extent and number of inflammatory cells. Mild lesions were those in which there were small and often multifocal accumulations of small numbers of inflammatory cells with minimal disruption of parenchyma. Moderate lesions were multifocal to more widespread, had moderate accumulations of inflammatory cells, and moderate disruption of parenchyma. Severe lesions had regional to diffuse accumulations of large numbers of inflammatory cells that often completely obscured or disrupted parenchyma. The distribution of the inflammation was defined as bronchopneumonia, bronchointerstitial pneumonia, or interstitial pneumonia. The types of inflammatory cells present within the inflammatory lesions were denoted as neutrophilic, eosinophilic, lymphoplasmacytic and granulomatous and were not mutually exclusive. Lesions were evaluated for the visual presence of bacterial and fungal organisms.

The cause of inflammation was defined as primary bacterial if bacterial organisms were visualized and associated with lesions and there was no concurrent evidence of lungworm, fungal or viral infection contributing to the inflammation. Inflammation was defined as primary lungworm if lesions were consistent with dolphin lungworm infection as previously described [25] and there was no concurrent or secondary bacterial, fungal, or viral infection. Inflammation was defined as primary fungal or viral-associated if the pathogen was visualized without other concurrent pathogens. Mixed infections were those in which there were multiple infectious agents (e.g. bacteria and lungworm) associated with the inflammatory lesions.

Pulmonary fibrosis was graded based on extent and frequency of lesions. Mild lesions were those in which lesions had a patchy distribution (less than 30% of the parenchyma was affected), and alveolar septae and/or perivascular connective tissue were mildly thickened by increased amounts of collagenous matrix. Moderate lesions were those which had a more widespread distribution (30–60% of parenchyma affected), alveolar septae and/or perivascular connective tissue moderately thickened by increased amounts of collagenous matrix, and/or thick bands of fibrous connective tissue with moderate disruption of pulmonary parenchyma. Severe lesions had large areas of parenchyma affected (greater than 60%), marked thickening of alveolar septae and/or perivascular connective tissue by increased amounts of collagenous matrix and large areas in which normal architecture was completely obliterated by fibrous tissue. Angiomatosis was defined as a proliferation of small thick-walled vessels, similar to lesions previously described at a prevalence of 46% in dolphins stranded in Texas from 1991 to 1996 [26]. Lungworm-associated pulmonary granulomas, when present, were defined as active or chronic similarly to what has been previously described in GoM dolphins [25].

#### Other histologic evaluations

Degree of inflammation in the central nervous system was subjectively graded as mild, moderate, or severe based on extent and number of inflammatory cells and frequency of inflammatory foci in tissues collected from the brain or spinal cord. Lymphoid depletion was evaluated as present or absent in lymph nodes and the spleen. Thymus was not available for examination in most cases, thus was not scored. Fibrosis, inflammation, necrosis, lipidosis, hemosiderosis, and biliary hyperplasia were evaluated in the liver. To assess nutritional status, presence or absence of thin epicardial adipose tissue, pancreatic zymogen depletion, and retained gastric superficial epithelial cell layers was determined in UME dolphins. The presence or absence of suspected morbillivirus infections or neurobrucellosis was determined based on available diagnostic tests and the presence or absence of representative histologic lesions in pulmonary, lymphoid, and central nervous system tissues similar to lesions previously described [16, 17, 27].

### Cause of death

Causes of death for UME and reference dolphins were acquired from original histopathology reports generated by a veterinary pathologist. Causes of death were based on histologic findings, gross necropsy reports, and ancillary test results. To ease comparisons among groups in the current study, causes of death were grouped into nine general categories: infectious, unknown—poor body condition, unknown—body condition not noted as likely contributor, trauma, organ failure, food obstruction/fish spine injury, maternal separation, and neoplasia. Cause of death was listed as multifactorial if more than one primary cause of death was noted by the pathologist. The prevalence of cause of death categories were determined for all UME dolphins, the Barataria Bay UME dolphin subset, UME dolphins with thin adrenal gland cortices, UME dolphins with primary bacterial pneumonia, all reference dolphins, and the standardly scored reference dolphin subset.

### Diagnostic tests

Morbillivirus, marine biotoxin, and *Brucella* tests were conducted on UME dolphins when appropriate tissues were available. Lung or lung-associated lymph node tissues, frozen and stored at -80°C, were tested for dolphin morbillivirus using a previously validated PCR assay [16]. Liver, feces, or gastric samples were analyzed for brevetoxin using an enzyme-linked immunosorbent assay (ELISA) and/or liquid chromatography—mass spectrometry (LC-MS), and analyzed for the presence of domoic acid by tandem mass spectrometry coupled with LC-MS/MS (MS) [12, 28, 29]. A *Brucella* PCR assay was used to test for *Brucella* in multiple tissues including lung, lung-associated lymph node, reproductive tract and/or central nervous system tissues frozen and stored at -80°C. Numbers and types of tissues tested varied by individual and were based upon tissues that were collected and available for testing. *Brucella* DNA was extracted from tissue samples using a commercially available kit (DNeasy, Qiagen) following the manufacturer’s instructions. Samples were tested for *Brucella* DNA using a semi-quantitative real-time PCR with standard reagents (Gene Expression Master Mix, Applied Biosystems) and a minor groove binding probe that cross-reacts with known terrestrial and marine *Brucella* species (University of Illinois, Zoological Pathology Program Molecular Diagnostic Laboratory). Presence of *Brucella* DNA was confirmed in all equivocal and positive samples by PCR amplification and sequencing of a 135 bp fragment of the *Brucella* 16SrRNA gene and/or sections of the outer membrane protein (OMP) 2 gene according to published protocols [30]. Nucleotide sequences were determined in both directions on an automated sequencer (Applied Biosystems 3730XL).

As a follow up to UME dolphins in which bacterial pathogens were suspected in the lung, lung samples from five dolphins were tested for the presence of bacterial pathogen DNA using a commercially available kit (MicroSeq 500 16S rDNA Bacterial Identification System, Life Technologies, Grand Island, NY, USA) according to the manufacturer’s instructions and compared nucleotide sequences using the MicroSeq ID Analysis Software. Lung samples from UME dolphins were also tested for the presence of *Nocardia* DNA using previously published protocols [31]. Necropsy culture swabs and tissues were also collected for classical microbial culture techniques. Isolated bacteria from lesions that were not believed to be due to overgrowth contamination, based upon the pathologists’ interpretation of tissues, were reported.

### Data management

A Microsoft Excel datasheet was used to enter the study data in a standardized way into an analyzable format. Variables in the datasheet included the unique animal field identifier, total body length (cm), sex, and state, county, and date of observed dolphin stranding. Diagnostic test results, including samples tested for morbillivirus, *Brucella*, and biotoxins were included. Histology variables described in the section above were entered into the database using standardized terms.

### Statistics

Data were analyzed using World Programming System (WPS 3.1) (World Programming Ltd., United Kingdom). The following four study groups were used for analysis: all UME dolphins, Barataria Bay subset UME dolphins, all reference dolphins, and the standardly scored subset reference dolphins. Descriptive statistics included stranding observation dates and state, sex, and body length. Body lengths and sex distribution were compared between UME and reference dolphins using a Wilcoxon rank-sum test and chi-square test, respectively. Throughout the UME and reference dolphin comparisons, sample sizes varied based upon information available, tissues collected, and tests conducted on individual UME and reference dolphins.

The prevalence of targeted histologic lesions and causes of death, described above, were compared between UME and reference dolphins using chi-square tests and odds ratios or Fisher’s exact tests (if compared categories had less than or equal to five values). To further assess the potential influence of sex and body length on those lesions with significant differences between standardly scored cases and references, a general linear model was used which included sex and body length as covariates and dummy, binary variables for the presence or absence of lesions. Significance was defined as a *P* value less than 0.05.

## Results

### UME and reference dolphin demographics

Forty-six fresh dead dolphins that stranded between June 2010 and December 2012 were included in the study (Table 1). A total of 106 reference dolphins were fresh dead and had tissues and/or histologic reports available, including dolphins that stranded along the Gulf coast of Florida (2002–2009), North Carolina (2004), South Carolina (1996–2012) and Texas (2003–2005). There were no significant differences in body length or sex when comparing GoM UME and reference dolphins (Table 2). Using a general linear model, body length and sex were reconfirmed as non-significant covariates for lesions with significant differences between UME and reference dolphins. Accordingly, potential physiologic influences of age and sex did not confound interpretation.

**Table 2:** Prevalence comparisons of lesions detected using histologic examination for fresh dead, stranded common bottlenose dolphins (*Tursiops truncatus*) among 1) UME cases from Louisiana, Mississippi, and Alabama, 2) Barataria Bay, Louisiana UME subset, 3) all reference dolphins, and 4) slide-reviewed and standardly scored reference dolphin subset.

Description	UME case dolphins All (n = 46)	Reference dolphins Standardly scored subset (n = 51)	*P* value (UME v. standardly scored reference dolphins)	UME case dolphin subset Barataria Bay only (n = 22)	Reference dolphins All (n = 106)
Body length (cm)	213 ± 37	225 ± 40	0.06	215 ± 39	211 ± 42
Female	19/46 (41%)	22/50 (44%)	0.79	9/22 (41%)	47/105 (45%)
Adrenal gland cortex					
Adrenal gland cortex (average C:M ± SD)	0.55 ± 0.23	0.60 ± 0.15	0.33	0.51 ± 0.22	0.60 ± 0.15
Thin adrenal gland cortex (C:M < 0.447)	12/36 (33%)	3/44 (7%)	0.003	9/18 (50%)^1^	3/44 (7%)
Thick adrenal gland cortex (C:M > 0.838)	3/36 (8%)	4/44 (9%)	0.91	1/18 (6%)	4/44 (9%)
Splenic lymphoid and lymph nodes					
Splenic lymphoid depletion	10/41 (24%)	3/42 (7%)	0.03	4/21 (19%)	13/73 (18%)
Lymph node depletion	10/40 (25%)	0/42 (0%)	< 0.0001	4/20 (20%)^1^	13/80 (16%)
Splenic lymphoid & lymph node depletion	5/35 (14%)	0/35 (0%)	0.05	1/19 (5%)	9/56 (16%)
Reactive lymph nodes	17/39 (44%)	11/36 (32%)	0.25	9/20 (45%)	20/67 (30%)
Liver					
Abnormal liver tissue	34/42 (81%)	40/45 (89%)	0.30	19/21 (90%)	76/94 (81%)
Fibrosis	29/42 (69%)	30/45 (67%)	0.82	17/21 (81%)	48/94 (51%)
Necrosis	2/42 (5%)	1/45 (2%)	0.61	2/21 (10%)	2/94 (2%)
Hepatitis	15/42 (36%)	23/45 (51%)	0.15	8/21 (38%)	41/94 (44%)
Hemosiderosis	13/42 (31%)	12/45 (27%)	0.61	8/21 (38%)	17/94 (18%)
Lipid deposition	5/42 (12%)	8/45 (18%)	0.44	4/21 (19%)	19/94 (20%)
Hepatobiliary hypertrophy	11/42 (26%)	12/45 (27%)	1.00	6/21 (29%)	24/94 (26%)
Central nervous system					
Abnormal CNS tissue	9/45 (20%)	13/44 (30%)	0.30	3/21 (14%)	32/85 (38%)^2^
Encephalitis^3^	9/45 (20%)	9/44 (20%)	0.96	3/21 (14%)	16/85 (19%)
Bacterial	5/45 (11%)	4/44 (9%)	1.00	2/21 (10%)	7/85 (8%)
Fungal	2/45 (4%)	1/44 (2%)	0.51	0 (0%)	3/85 (4%)
Parasitic	1/45 (2%)	2/44 (5%)	0.62	0 (0%)	4/85 (5%)
Viral	1/45 (2%)	5/44 (11%)	0.11	0 (0%)^1^	5/85 (6%)^2^
*Brucella* PCR-positive CNS tissue	2/33 (6%)	0/2	NA	1/14 (7%)	1/13 (8%)
Morbillivirus PCR-positive CNS tissue	5/33 (15%)	Not tested	NA	1/14 (7%)	Not tested

Denominators varied based upon information available, tissues collected from, and tests conducted on individual UME and reference dolphins.

^1^Barataria Bay, Louisiana UME dolphin subset values different (*P* < 0.05) than standardly scored reference dolphins.

^2^UME dolphin values different (*P* < 0.05) than full reference group.

^3^Represents encephalitis, meningitis, or meningoencephalitis.

C:M = adrenal corticomedullary ratio. CNS = central nervous system.

### Adrenal gland

**Fig 1:**
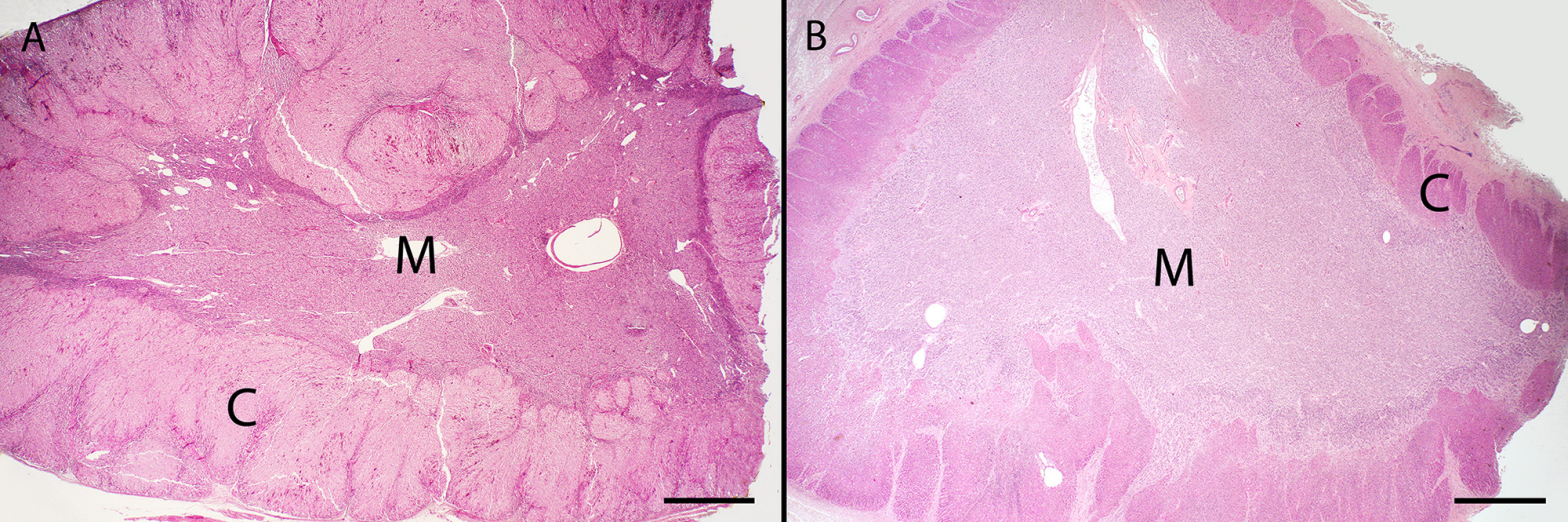
Hematoxalin and eosin stained sections of adrenal glands from common bottlenose dolphins (*Tursiops truncatus*) C = cortex; M = medulla. A. Normal adrenal gland from a subadult male dolphin that stranded fresh dead along the west coast of Florida in 2003. Bar = 1 mm. B. Adrenal gland from a subadult male dolphin that stranded in Barataria Bay, Louisiana in 2011. Note the thin adrenal cortex, especially in the fasciculata region. Bar = 1mm.

Among UME dolphins in which appropriate adrenal gland tissue was available for evaluation, one-third (12/36) had a thin adrenal gland cortex and low corticomedullary ratio (less than 0.447), including 9 (50%) Barataria Bay dolphins (Table 2). UME dolphins were more likely to have a thin adrenal gland cortex compared to reference dolphins (33% *versus* 7%, *P* = 0.003, Table 2). Adrenal glands with a thin cortex had reduced numbers of cells with only a thin band of cortex remaining (Fig 1). In most cases, distinction between the zona fasciculata and reticularis was difficult. Although cells in the zona fasciculata region appeared reduced in number, it was not possible to distinguish whether the zona glomerulosa and zona reticularus were also affected. In the most severely affected cases, corticocytes were small, though cell size varied greatly within and among the cortex of all individuals. Some dolphins without a thin cortex had small corticocytes. There was no evidence of hemorrhage, necrosis, or fibrosis in affected adrenal glands. The size of the adrenal medulla was within normal limits for all dolphins.

**Fig 2:**
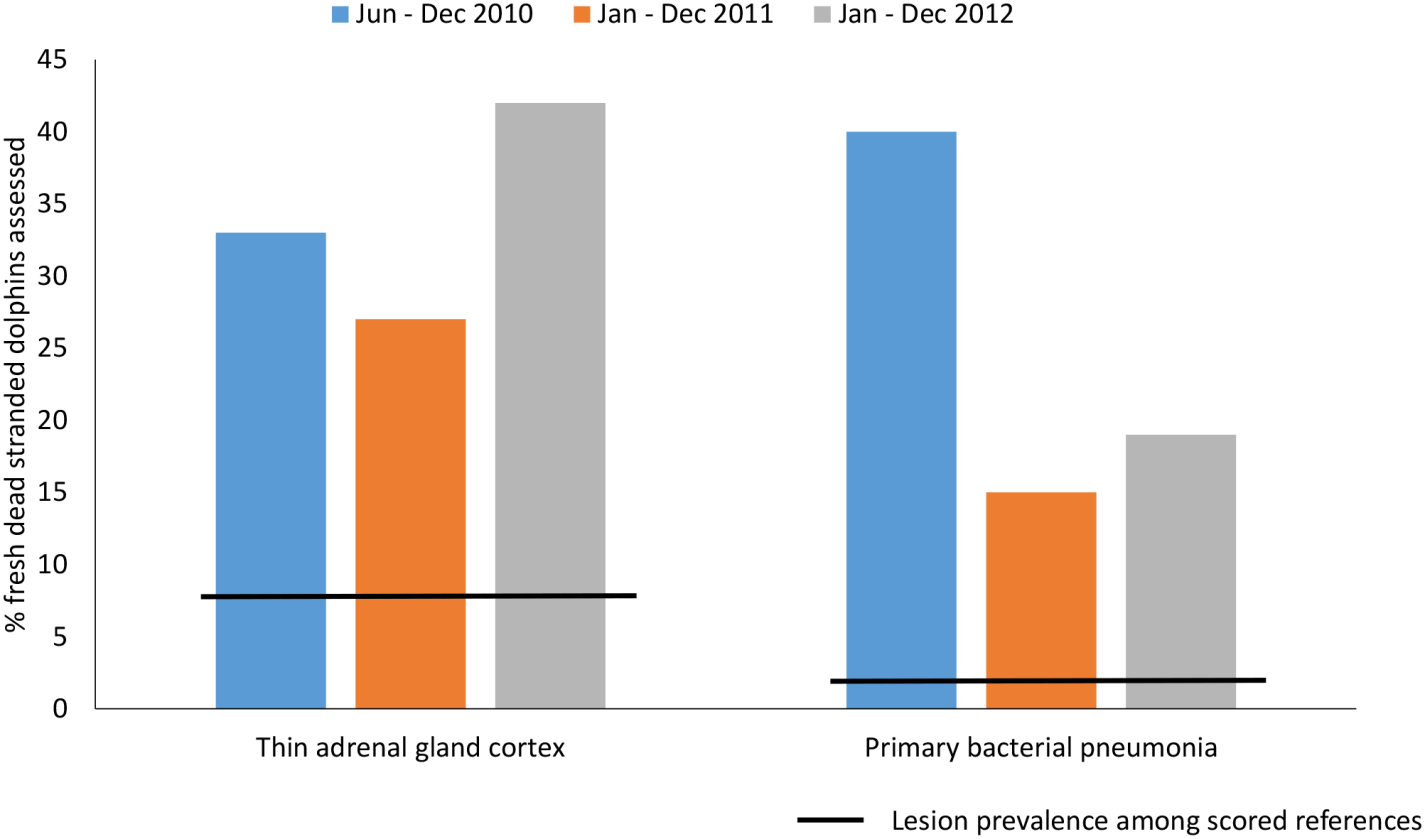
Prevalence of key histologic lesions among fresh dead, stranded common bottlenose dolphins (*Tursiops truncatus*) by year in Louisiana, Mississippi & Alabama: June 2010 to December 2012. The gray line represents the percentage of lesion prevalence among standardly scored reference dolphins. All years had prevalence of lesions greater than the reference group (*P* > 0.05).

The proportion of stranded dolphins with thin adrenal gland cortices was similarly high by year (June–December 2010 = 3/9, 33%, 2011 = 4/15, 27%, 2012 = 5/12, 42%) (Fig 2). Of UME dolphins with a thin cortex, 4 (33%) had a concurrent primary bacterial pneumonia. None of the thin adrenal cortex UME dolphins had evidence of morbillivirus infection, and one case had *Brucella* meningoencephalitis. None of the UME dolphins with a thin cortex had depleted epicardial adipose tissue, an indicator of advanced, poor nutritional state. The most common noted cause of death among dolphins with a thin adrenal cortex was unknown death not attributed to poor body condition (42%).

Among all UME dolphins, mild adrenal corticocyte vacuolation was noted in three (7%) dolphins, and adrenal gland inflammation was noted in two (4%); one of these dolphins had intralesional bacteria associated with disseminated infection. There was no significant difference in the prevalence of corticocyte vacuolation between UME and reference dolphins (7% *versus* 12%, *P* = 0.49). No other pathogens were noted in any other UME dolphin adrenal glands.

### Lung

Of the UME dolphins, 22% had a primary bacterial pneumonia. In 7 (70%) of UME dolphins with primary bacterial pneumonia, the condition either caused or contributed significantly to death (Tables 3 and 4). The prevalence of primary bacterial pneumonia was higher than reference dolphins (2%) (*P* = 0.003). Of the 10 UME dolphins with primary bacterial pneumonia, 5 (50%) had severe pneumonia. Overall, UME dolphins had a higher prevalence of severe pneumonia compared to standardly scored references (16% *versus* 2%, *P* = 0.03). Primary bacterial pneumonia was most common in UME dolphins from June to December 2010 and was less prevalent during 2011 and 2012 (2010 = 4/10, 40%, 2011 = 3/20, 15%, 2012 = 3/16, 19%) (Fig 2). All UME years examined, however, had a higher prevalence of primary bacterial pneumonia compared to reference dolphins (Fig 2, line indicating prevalence among standardly scored reference dolphins). Six (60%) UME dolphins with primary bacterial pneumonia had bronchopneumonia. UME dolphins overall had a higher prevalence of inflammatory lung infiltrate characterized as granulomatous compared to the standardly scored reference dolphins (78% versus 57%, *P* = 0.03, Table 3).

**Table 3:** Prevalence comparisons of lung lesions detected using histologic examination for fresh dead, stranded common bottlenose dolphins (*Tursiops truncatus*) among UME dolphins from 1) Louisiana, Mississippi, and Alabama, 2) slide-reviewed and standardly scored reference dolphin subset, 3) Barataria Bay, Louisiana UME dolphin subset, 4) and all reference dolphins.

Description	UME case dolphins All(n = 46)	Reference dolphins Standardly scored subset (n = 51)	*P* value (UME v. standardly scored reference dolphins)	UME case dolphin subset Barataria Bay only (n = 22)	Reference dolphins All (n = 106)
Abnormal lung	46 (100%)	51 (100%)	1.00	22 (100%)	104 (98%)
Pneumonia	44 (96%)	43 (84%)	0.07	20 (91%)	88 (83%)^2^
Severe	7 (15%)	1 (2%)	0.03	1 (5%)	6/86 (7%)
Distribution:					
Bronchopneumonia	30 (65%)	23 (45%)	0.05	16 (73%)^1^	33/80 (41%)^2^
Interstitial pneumonia	4 (9%)	5 (10%)	0.57	2 (9%)	26 (33%)
Bronchointerstitial	10 (22%)	15 (29%)	0.39	2 (9%)	21 (26%)
Inflammatory infiltrate:					
Granulomatous	36 (78%)	29 (57%)	0.03	18 (82%)	37/73 (51%)^2^
Lymphoplasmacytic	26 (57%)	23 (45%)	0.26	13 (59%)	37 (51%)
Eosinophilic	13 (28%)	13 (25%)	0.76	7 (32%)	27 (37%)
Suppurative	25 (54%)	18 (35%)	0.06	12 (55%)	19 (26%)^2^
Cause:					
Primary lungworm	24 (52%)	31 (61%)	0.14	13 (59%)	65/84 (77%)^2^
Primary bacterial	10 (22%)	1 (2%)	0.003	4 (18%)^1^	2 (2%)^2^
Primary viral	4 (9%)	1 (2%)	0.19	1 (5%)	1 (1%)
Primary fungal	0 (0%)	1 (2%)	NA	0 (0%)	1 (1%)
Primary protozoal	0 (0%)	0 (0%)	NA	0 (0%)	1 (1%)
Mixed	4 (9%)	10 (20%)	0.16	2 (9%)	14 (17%)
Aspiration and secondary bacterial	1 (2%)	0 (0%)	NA	0 (0%)	0 (0%)
Pathogens identified:					
Lungworm	19 (41%)	37 (73%)	0.002	10 (45%)^1^	66/106 (62%)^2^
Bacteria	15 (33%)	8 (16%)	0.05	6 (27%)	14/102 (14%)^2^
Virus	4 (9%)	1 (2%)	0.18	1 (5%)	2/102 (2%)
Fungus	3 (7%)	3 (6%)	0.71	0 (0%)	3/102 (3%)
Fibrosis	40 (87%)	40 (78%)	0.27	20 (91%)	57 (54%)^2^
Moderate to severe	16 (35%)	17 (33%)	0.89	7 (32%)	30 (28%)
Angiomatosis	24 (52%)	31 (61%)	0.39	12 (55%)	46/59 (78%)^2^
Granuloma	16 (35%)	19 (37%)	0.80	9 (41%)	31 (29%)
Active	7 (15%)	5 (10%)	0.42	3 (14%)	14 (13%)
Chronic	9 (20%)	13 (25%)	0.68	6 (27%)	16 (15%)
Lung or lung-associated lymph node *Brucella* PCR-positive	2/38 (5%)	1/12 (8%)	0.58	1/20 (5%)	3/37 (8%)
Lung morbillivirus PCR-positive	6/43 (14%)	Not tested	NA	2/20 (10%)	Not tested

Denominators varied based upon information available, tissues collected from, and tests conducted on individual UME and reference dolphins.

^1^Barataria Bay, Louisiana UME dolphin subset values different (*P* < 0.05) than standardly scored references;

^2^All reference dolphin values different (*P* < 0.05) than all UME dolphins

**Table 4:** Prevalence comparisons of causes of death for fresh dead, stranded common bottlenose dolphins (*Tursiops truncatus*) among 1) unusual mortality event cases from Louisiana, Mississippi, and Alabama, 2) slide-reviewed and standardly scored reference dolphin subset, 3) Barataria Bay, Louisiana UME dolphin subset, and 4) all reference dolphins.

Description	UME case dolphins all (n = 46)	References standardly scored set (n = 36)	*P* value (UME v. standardly scored reference dolphins)	UME cases Barataria Bay (n = 22)	Thin adrenal cortex cases (n = 12)	Primary bacterial pneumonia cases (n = 10)
Infection	18 (39%)	5 (14%)	0.01	6 (27%)	3 (25%)	7 (70%)*
Unknown	13 (28%)	9 (25%)	0.84	9 (41%)	5 (42%)	0
Unknown—poor body condition	3 (7%)	3 (8%)	0.54	2 (9%)	0	0
Unknown—body condition not noted likely contributor	10 (22%)	6 (17%)	0.57	7 (32%)	5 (42%)	0
Trauma	8 (17%)	10 (28%)	0.26	3 (14%)	1 (8%)	0
Multifactorial	4 (9%)	2 (6%)	0.69	2 (9%)	2 (17%)	3 (30%)
Organ failure	2 (4%)	0	NA	1 (5%)	1 (8%)	0
Food obstruction/spine injury	1 (2%)	8 (22%)	0.005	1 (5%)	0	0
Maternal separation	0	2 (6%)	NA	0	0	0
Neoplasia	0	0	NA	0	0	0

Denominators varied based upon information available, tissues collected from, and tests conducted on individual UME and reference dolphins.

*Values different (*P* < 0.05) than reference dolphins.

**Fig 3:**
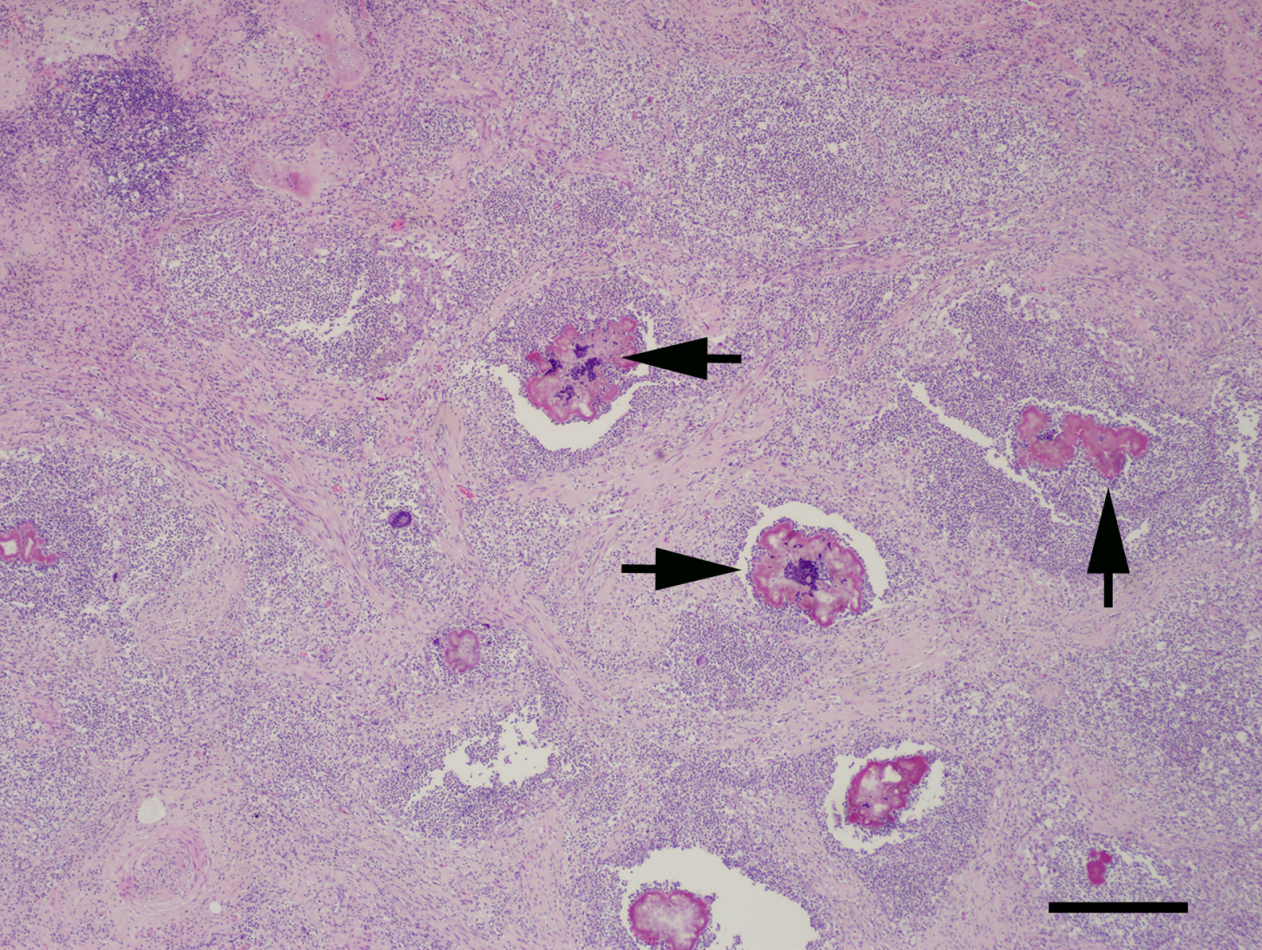
Hematoxalin and eosin (HE) stained section of lung from an adult female common bottlenose dolphin (*Tursiops truncatus*) that stranded in Barataria Bay, Louisiana in December 2011 with primary bacterial pneumonia. Airspaces and septae are obscured by accumulations of neutrophils and fibrous connective tissue that surround large bacterial colonies (arrows). Bar = 500 μm.

In UME dolphins with primary bacterial pneumonia, bronchioles and alveolar spaces were often filled with viable and necrotic neutrophils (i.e. suppurative) and fewer macrophages. Bacteria were extracellular or intracellular within neutrophils and macrophages. In some cases, there was extensive necrosis. In the most severe cases, large accumulations of inflammatory cells completely obscured normal pulmonary parenchyma. Inflammatory cells and bacteria were encircled by dense bands of fibrous connective tissue and there were multifocal abscesses (Fig 3). In several cases, inflammatory cells surrounded bacterial colonies that were bordered by radiating, bright eosinophilic club-shaped material (Splendore-Hoeppli material).

Among UME dolphins with primary bacterial pneumonia, bacteria within lung lesions included coccobacilli (n = 3), cocci (n = 2), and gram positive acid fast negative filamentous bacteria (n = 2). Pulmonary *Escherichia coli*, *Staphylococcus aureus*, and *Streptococcus* spp. Group G infections were confirmed via bacterial culture of lung tissue in UME cases. *Staphylococcus aureus* and *Streptococcus spp*. infections were associated with sepsis. Another UME dolphin with primary bacterial pneumonia had concurrent PCR-confirmed *Brucella* meningoencephalitis, although *Brucella* PCR on lung tissue was negative.

Among UME dolphins overall, two dolphin lungs were positive for *Brucella* sp. via PCR. Histologically, these dolphins did not have lung lesions consistent with active brucellosis. Additional bacterial PCR assays used in other primary bacterial pneumonia cases were unsuccessful in amplifying any single pathogenic bacterial species from affected lung tested, likely due to postmortem bacterial overgrowth.

Of UME dolphins with primary bacterial pneumonia and had a measurable adrenal gland, 4n/9 (44%) had a thin adrenal gland cortex. One of 10 (10%) UME dolphins with primary bacterial pneumonia and known cardiac histologic evaluation had depleted cardiac adipose tissue, 3 of 6 (50%) had zymogen depletion, and 2 of 6 (33%) had retained gastric epithelium. Other common pulmonary lesions among UME and reference dolphins included lungworm pneumonia, pulmonary fibrosis, and angiomatosis. The most common cause of pneumonia amongst both UME and reference dolphins was primary lungworm infection (Table 3). Reference dolphins, however, were more likely to have this common cause of pneumonia than the UME dolphins (62% *versus* 41%, respectively, *P* = 0.005).

### Lymphoid tissues

UME dolphins had a higher prevalence of lymphoid depletion in either or both the spleen or lymph node than reference dolphins (Table 2). When lymphoid depletion was present in either the lymph nodes or the spleen, both B lymphocyte (lymphoid follicles in the spleen and lymph node and medullary cords of the lymph node) and T lymphocyte (periarterior lymphoid sheaths in the spleen and the paracortex of the lymph nodes) regions were affected in the majority of the UME cases. Affected lymphoid follicles had a thin mantle zone often depleted of small lymphocytes. Of the five UME cases with both splenic and lymph node depletion, three died from morbillivirus infections, one died from non-*Brucella* bacterial meningoencephalitis, and another died from generalized debilitation associated with poor body condition.

### Central nervous system

Meningitis or meningoencephalitis were equally common in UME and reference dolphins (20% and 30%, respectively) (Table 2). Central nervous system (CNS) inflammation among UME dolphins was characterized as mild (n = 1), moderate (n = 5), and severe (n = 3). The most common pathogen category associated with meningoencephalitis was bacterial (5, 11% of UME dolphins), which was similar to the reference dolphins (4, 9%) (Table 2). Three (7%) UME dolphins had *Brucella-*associated meningoencephalitis. Two of the UME dolphins with encephalitis or meningoencephalitis had a thin adrenal gland cortex, and one had a primary bacterial pneumonia.

### Liver

**Fig 4:**
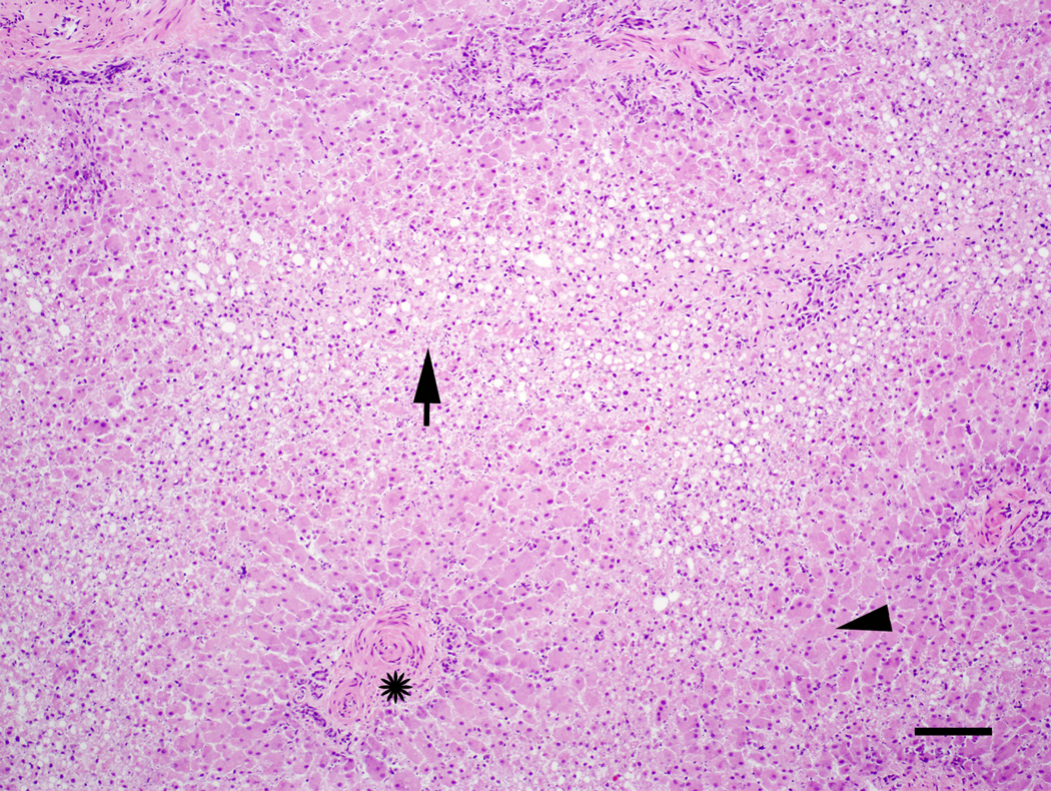
Hematoxylin and eosin (HE) stained section of liver from a juvenile dolphin that stranded fresh dead in Barataria Bay in July 2012. There is severe centrilobular hepatocellular vacuolation, necrosis, and loss with lobular collapse (arrow). Star indicates a portal triad. Arrow head indicates unaffected liver parenchyma for comparison. Bar = 500μm.

The prevalence of hepatic lesions, including fibrosis, necrosis, hepatitis, hemosiderosis, lipid deposition, and hepatobiliary hypertrophy, was similar among UME and reference dolphins (Table 2). The prevalence of moderate to severe hepatic fibrosis (31% *versus* 36%, *P* = 0.65) and inflammation (2% *versus* 7%, *P* = 0.62) were also comparable between the UME and reference dolphins. Fibrosis and inflammation had a periportal distribution in the majority of the dolphins from both groups, and lesions were most often mild to moderate in severity. Two UME cases were identified with centrilobular hepatocellular vacuolation, degeneration, necrosis, and loss with stromal collapse (Fig 4). Both of these dolphins stranded in Barataria Bay, one in June 2011 and one in July 2012. The June 2011 stranded dolphin was found live and externally oiled. The July 2012 stranded dolphin had evidence of jaundice on gross examination consistent with significant liver disease.

### Causes of death

The most common cause of death for UME dolphins was infectious disease (39%), and UME dolphins were four times more likely to die from infectious causes than the reference dolphins (14%, *P* = 0.01) (Table 4). The most common causes of death for Barataria Bay dolphins were infectious disease (27%) and unknown, not attributed to poor body condition (32%) (Table 4). An unknown cause of death not attributed to poor body condition was most common in UME dolphins with a thin adrenal gland cortex (42%, 5/12). Only three of 19 (16%) UME dolphins that died from infectious causes (and had available adrenal gland for measurement) had a thin adrenal gland cortex; of all 19 UME dolphins that died from infectious causes, eight (42%) had a primary bacterial pneumonia, and five (26%) died from morbillivirus infections.

### Nutritional status

Of UME dolphins in which appropriate tissues were available to asses nutritional status, 2/42 (5%) had thin epicardial adipose tissue indicative of emaciation. There were also indicators of inanition by pancreatic zymogen depletion and retained superficial gastric epithelium in 5/28 (18%) and 7/27 (26%) of dolphins, respectively.

### Morbillivirus, *Brucella*, and marine biotoxins

A total of 6/43 (14%) lung and 5/33 (15%) CNS samples from UME dolphins were positive for morbillivirus using PCR, with a total of 8 (19%) UME dolphins with tissues positive for morbillivirus. Five of these eight UME cases had relevant histologic lesions in tissue from which morbillivirus was identified. Five UME morbillivirus PCR positive cases stranded in 2011 and three in 2012 in multiple states. Most (6 of 8) UME dolphins with morbillivirus infections were either calves, juveniles, or subadults. Lesions noted in morbillivirus-affected dolphins included splenic and lymph node lymphoid depletion, syncytial cells, pneumonia, and lymphocytic encephalitis. Two cases had concurrent fungal infections.

Three cases of brucellosis, all with moderate to severe *Brucella*-associated lymphoplasmacytic meningoencephalitis and confirmed identification of *Brucella* using PCR or culture, were identified among UME dolphins. The prevalence of brucellosis based upon histopathology and ancillary testing in UME dolphins (6%) was similar to those in the standardly scored subsets (2/42, 5%, *P* = 0.68). There were also no differences in the prevalence of *Brucella* detection using PCR on lung or lung-associated lymph node between UME and reference dolphins (5% and 8%, respectively, *P* = 0.58, Table 3). All UME dolphins with brucellosis were males.

Fecal, gastric content, or liver samples from twenty-eight of the UME dolphins included in this study were tested for brevetoxin and/or domoic acid. Of 26 UME dolphins tested for brevetoxin, one dolphin had detectible levels of brevetoxin by ELISA (2 ng/g) but was negative by LC-MS. Of 27 UME dolphins tested for domoic acid, three were positive at low concentrations (all were 8 ng/g). All positive results were consistent with background exposures [12].

## Discussion

To our knowledge, adrenal cortical atrophy as found in this study has not been previously described in free-ranging cetaceans, including bottlenose dolphins previously studied in the northern GoM [32]. The normal corticomedullary ratio of dolphin adrenal glands has been determined to be approximately 1:1 [24]. Thus, the discovered high prevalence of adrenal cortical atrophy in dolphins stranding during the ongoing GoM UME may be part of a syndrome that has not been previously reported in dolphins during mortality events. The prevalence of adrenal cortical atrophy identified in this study is consistent with the high prevalence (approximately 50%) of live Barataria Bay dolphins with evidence of hypoadrenocorticism assessed during 2011, including a relatively high proportion of dolphins with low blood cortisol, aldosterone, and glucose [11]. Follow up evaluation of adrenal glands from stranded dolphins in subsequent years will help to determine the persistence of adrenal insufficiency observed relative to the timing of the UME and the concurrent DWH oil spill.

There are a number of different causes of adrenal insufficiency in mammals, including autoimmune disease, metastatic neoplasia, fungal infections, stress, trauma, miliary tuberculosis, corticosteroid toxicity, and contaminant exposure [33]. Additionally, infection with phocine herpesvirus-1 has been demonstrated to cause adrenal cortical necrosis in marine mammals [34]. In the current study, only 2 of 46 UME dolphins had inflammation in the adrenal gland, and with the exception of one case with a disseminated bacterial infection, neither infectious agents nor neoplasia were identified in UME dolphin adrenal glands. Further, there was no histologic evidence of autoimmune adrenalitis or neoplasia in any UME dolphin adrenal glands, indicating that adrenal cortical atrophy in UME dolphins was not due to direct infection of the adrenal gland, autoimmune disease, or neoplasia.

In humans, chronic demand on the adrenal glands, including chronic illness, has been postulated to lead to cortical thinning and potential adrenal exhaustion associated with lipid depletion of the fasciculata cells [35,36]. Previous evaluations of adrenal glands from stranded GoM dolphins from Texas with both acute and chronic disease have been conducted, but no cases of adrenal cortical atrophy were identified [32]. Instead, adrenal glands of dolphins dying from chronic disease (likely chronically stressed individuals) were significantly heavier, and corticomedullary ratios were significantly higher than those dying from acute disease or acute trauma. Findings from Clark et al. (2006) suggest that adrenal gland enlargement and cortical hyperplasia are common responses to chronic stress and disease in bottlenose dolphins, similar to that noted in other cetaceans and other mammalian species [24, 32, 37–39]. Of the 12 UME dolphins with a thin adrenal gland cortex, one-third had primary bacterial pneumonia, leaving the majority of adrenal cortex cases without evidence of active or chronic infections. Further, none of the UME dolphins with a thin adrenal gland cortex had depleted cardiac adipose tissue, indicating that UME dolphins were not in an advanced, debilitated nutritional state. These results do not support general infection or chronic poor body condition as underlying causes of adrenal gland cortex depletion.

Although the effects of polycyclic aromatic hydrocarbon (PAHs) on the hypothalamus-pituitary-adrenal (HPA) axis are poorly understood, the adrenal gland is reported to be the most common endocrine organ to exhibit lesions with exposure to toxigenic chemicals [40, 41]. In general, mechanisms of direct adrenal toxicity include impaired steroidogenesis, activation of toxins by cytochrome p450 enzymes generating reactive oxygen metabolites, DNA damage, and exogenous steroid action [42]. The adrenal gland can be a significant site for metabolism of PAHs, thus increasing the adrenal gland to exposure from these contaminants and their metabolites [43].

Several studies have shown that PAHs or oil can affect the HPA axis and adrenal gland function. Hypoadrenocorticism has been reported in mink fed either bunker C or artificially weathered fuel oil [44,45]. In these mink, adrenal cortical hypertrophy with vacuolation of corticocytes was detected histologically. These studies, however, did not monitor changes in response to higher level exposure and/or over longer periods of time. Chemicals that induce adrenal cortical vacuolar degeneration can lead to loss of adrenocortical cells due to necrosis and adrenal cortical atrophy. It is possible PAHs may act in a similar fashion [42]. Naphthalene, a common PAH associated with crude oil, reduced plasma corticosterone in mallard ducks following ingestion of petroleum-contaminated food, and a similar acute decrease in cortisol was detected in exposed eels [46, 47]. House sparrows exposed orally to 1% crude oil from the GoM exhibited decreases in cortisol in response to stressors or to adrenocorticotropin hormone injection [48].

Mammalian exposure to PAHs can greatly increase hepatic metabolism of other compounds (e.g. 7,12-dimethylbenz(α)anthracene), which in turn can cause targeted and severe injury to the adrenal gland, including necrosis and hemorrhage [49–51]. Removal of the inciting chemical, if the adrenal cortical injury is not too advanced, may result in regained function and resolved lesions characterized by fibrosis, atrophy, nodular regeneration or calcification [42, 47, 51]. Ultrastructural analysis can be beneficial in helping to identify direct toxic damage. Unfortunately, optimally fixed, minimally autolyzed tissue from affected dolphin adrenal glands was not available for ultrastructural analysis. The lack of adrenal lesions beyond cortical atrophy suggests, however, that potential chemical effects may be higher in the HPA axis [52].

During and following the DWH oil spill, significantly elevated PAH levels were detected in the coastal GoM waters, including Louisiana, Mississippi, and Alabama [53]. These locations coincide with the states most impacted by the ongoing UME since the DWH oil spill [1]. Thus, northern GoM dolphins’ exposures to DWH spill-associated PAHs, especially in Louisiana and Mississippi, may account for the observed effects on adrenal function found in both live and dead dolphins [11]. Given the lack of evidence of alternative causes of adrenal cortical atrophy and the high prevalence of this lesion among stranded dolphins following the DWH oil spill, the leading hypothesis is that exposure to contaminants from the DWH oil spill led to chronic injury of the adrenal gland cortex at least through 2012.

Chronic adrenal insufficiency (CAI) is a life-threatening disease that can lead to adrenal crisis and death in mammals [53]. Adrenal crises in people with CAI are triggered by infections, fever, major pain, psychological distress, heat, and pregnancy [54]. Cold temperatures can also increase the risk of death among animals with CAI. Angora goats with a genetically-driven high incidence of primary CAI lack proper cortisol and glucose response and, as such, are susceptible to die-offs from cold stress [54, 55]. GoM dolphins were exposed to colder than normal temperatures during early 2011, and if those dolphins from the UME had pre-existing CAI, they may have been at higher risk of cold stress-related deaths [6]. This hypothesis is further supported in that dolphins have a compensatory adrenal response in cold temperatures, including increased cortisol levels, presumably to help generate metabolic heat [56]. Adrenal crisis may have been the cause of death for many of the UME dolphins with adrenal cortical atrophy following stress events to which a healthy dolphin could have otherwise adapted. In addition to the cold weather during 2011, adrenal crisis could have also been precipitated by late-term pregnancies and infections, including bacterial pneumonia [57].

Compared to reference dolphins, UME dolphins were more likely to have a primary bacterial pneumonia. Many of these pneumonias were much more severe than bacterial pneumonias in the reference dolphins. These findings are consistent with the high prevalence of moderate to severe lung disease detected in live Barataria Bay dolphins [11]. During the DWH oil spill and response period, numerous dolphins, including dolphins in Barataria Bay, were observed swimming through visibly oiled waters and feeding in areas of surface, subsurface, and sediment oiling [11]. As mentioned, the presence of increased coastal PAH levels associated with the DWH oil spill, especially near Grand Isle, Louisiana in Barataria Bay, have been confirmed, indicating an increased risk of inhaled PAHs in dolphins [4]. Given that the dolphin's blowhole is at the surface of the water, chemicals, including volatile PAHs, could have been readily inhaled. In other animals, inhaled PAHs can irritate airways, denude mucosal surfaces, and cause peribronchial inflammation and systemic toxicity [58]. Damaged epithelium and cilia, in turn, can severely impair immune defenses.

In other animals, the severity of chemical inhalation injury is dependent on breathing patterns, in which deep breaths increase injury to tissues deeper within the lung [59]. This pattern is of particular importance given the dolphin's respiratory anatomy and physiology. While humans exchange approximately 10 to 20% of air with each breath, dolphins exchange 75 to 90% of deep lung air [60–63]. They also lack nasal turbinates and cilia to filter the air prior to reaching the lungs, and have deep inhalations followed by a breath hold that provides potential for more prolonged contact and exchange between air-borne particulates and the blood [60–63]. All of these factors would likely amplify the effects of inhaled chemical irritants in dolphins compared to observations and studies involving other mammals.

The severe bacterial pneumonias found in UME dolphins could represent a chronic sequelae to hydrocarbon inhalation or aspiration, or have been secondary to PAH induced immune system compromise. The most common sequela to hydrocarbon inhalation and ingestion in humans and animals are aspiration pneumonia and pneumonia often involving the bronchioles [64–68]. Inhaled hydrocarbon vapors or aspirated hydrocarbons may cause necrosis of bronchial and bronchiolar epithelium, and pneumocyte and alveolar septal necrosis which leads to inflammation and secondary infection [64–66]. During the 2007 firestorm in San Diego, dolphins and people living in San Diego Bay area were exposed to high levels of PAHs [69, 70]. The month following the fires, these dolphins demonstrated decreased absolute and percent neutrophils [70]. This change indicated that dolphins exposed to PAHs through inhalation may have had a compromised immune response and an increased risk of acquiring bacterial pneumonia.

In addition to inhalation risks, hydrocarbon ingestion can lead to gastrointestinal mucosal irritation, vomiting or regurgitation, and resultant aspiration pneumonia. Cattle that ingest petroleum develop bacterial pneumonia due to chemical-induced regurgitation and/or aspiration [67,68]. Correspondingly, based on histologic examination, one dolphin that stranded during June 2010 in Mississippi during the DWH oil spill had suspected aspiration pneumonia, secondary bacterial infection, ulcerative tracheitis, and ulcerative gastritis with edema. Both the tracheal and gastric lesions, although non-specific, could have resulted from mucosal irritation, such as may occur with toxin ingestion.

Though there was no difference in prevalence of liver lesions when comparing UME and reference dolphins in this study, two UME dolphins had similar severe centrilobular liver lesions characterized by hepatocyte loss, necrosis and vacuolation that could potentially be associated with toxin exposure. Both of these dolphins stranded in Barataria Bay. Hepatocellular vacuolation, degeneration and necrosis have been associated with exposure to crude oil and benzo[a]pyrene (BaP) [71 72]. Hepatotoxic liver injury may occur due to xenobiotic metabolism of substances producing injurious metabolites and lesions most often occur in the centrilobular zones where hepatocytes have the highest concentration of cytochrome p450 enzymes [71]. Other rule-outs for centrilobular degeneration and necrosis include hypoxia, severe or precipitous anemia (e.g. hemolytic anemia), chronic heart disease, or circulatory failure associated with septic shock [72, 73]. There was no other evidence of hypoxia, hemolytic anemia or heart disease in either of the affected dolphins and lesions were more chronic than would be expected secondary to acute hypoxia or shock. Some oiled sea otters that died following the Exxon Valdez oil spill had centrilobular hepatic necrosis, though whether the lesions were due to direct toxic insult or secondary to anemia is unclear [74]. Biliary or periportal inflammation and fibrosis secondary to infection by the trematode *Campula spp*. are common hepatic lesions noted in a number of cetacean species, and periportal lesions noted in both UME and reference dolphins were consistent with the chronic sequelae of biliary trematode infection [75].

This study did not support that previously documented or suspected contributing factors for GoM dolphin UMEs were primary contributors to the ongoing UME among non-perinatal dolphins. All UME dolphins in this case study had biotoxin levels that were below detectable levels except for one with low levels [12]. Relatively few morbillivirus cases were identified among UME cases. In previous known dolphin morbillivirus-associated die-offs, more than 60% of cases tested positive for the virus when using a similar PCR assay [14,16]. Exposure to morbillivirus has been documented in GoM dolphins, and the cases identified in 2011 and 2012 may represent exposure to the virus in a small number of susceptible individuals in the population [76]. Similarly, there were too few brucellosis cases in this study to explain the ongoing UME, with only two cases that had *Brucella* identified in the lung, demonstrating that *Brucella* was not the driver for increased primary bacterial pneumonia. Despite global reports of *Brucella* infections in marine mammals, there have been, to date, no documented brucellosis epizootics in cetaceans [17].

Due to the long duration and large scope of the ongoing UME, there may be multiple factors affecting the health of dolphins by region through time. Aside from the DWH oil spill, there were two relatively smaller oil spills that occurred in and around Barataria Bay during this study’s timeframe. Specifically, the T/V Pere Ana C spill in Mud Lake, Louisiana on July 27, 2010 (approximately 7,000 gallons spilled) and the Cedyco Manilla Village Spill in Bayou Dupont, Louisiana which occurred on September 11, 2011 (approximately 10,500 gallons) [77,78]. In comparison, however, the DHW oil spill released approximately 126,000,000 gallons and was visible across 40 km and 366,000 m^2^ of Barataria Bay’s shoreline in decreasing amounts over time for at least 2 years, demonstrating the higher magnitude of the DWH oil spill’s likely impact compared to other spills [7–10].

The Gulf of Mexico has historically had documented dead zones with episodes of seasonal hypoxia associated with nutrient loading from the Mississippi River watershed [79,80]. The geographic area and clusters of dolphin stranding identified from the current UME, however, were not limited to single seasons or specific dead zone hotspots [2]. Further, dead zones are often associated with fish die-offs and habitat loss; the lack of emaciation as the primary contributor to the deaths of dolphins in this study supports that the primary driver of this UME was not loss of prey [80].

The lack of baseline diagnostic and histologic data on fresh stranded dolphins prior to 2010 in the UME area, as well as during the pre-DWH oil spill period, paired with an assumption that stranded dolphins should have similar lesion prevalence regardless of location, are limitations in this study. The surprisingly high number of assessed lesions that were not significantly different between the UME and reference dolphins, however, increased the confidence that the study groups were indeed comparable. Continual assessment of trends and changing disease states over time are needed, however, to better understand the potential roles of multiple contributing factors to dolphin mortality during the ongoing UME.

In summary, UME dolphins had a high prevalence of thin adrenal gland cortices (especially in Barataria Bay dolphins) and primary bacterial pneumonia. These findings are consistent with endocrinologic and pulmonary-based observations of live bottlenose dolphins from health assessments in Barataria Bay during 2011 [11]. Previously documented or suspected contributing factors for GoM UMEs (marine biotoxins, morbillivirus, and brucellosis) were not supported by this study as contributors to the ongoing UME. Due to the timing and nature of the detected lesions, we hypothesize that contaminants from the DWH oil spill contributed to the high numbers of dolphin deaths within this oil spill’s footprint during the northern GoM UME following the DWH oil spill. Direct causes of death likely included: 1) affected adrenal gland cortices, causing chronic adrenal insufficiency, 2) increased susceptibility to life-threatening adrenal crises, especially when challenged with pregnancy, cold temperatures, and infections, and 3) increased susceptibility to primary bacterial pneumonia, possibly due to inhalation injury, aspiration of oil, or perturbations in immune function.

## Supporting Information

S1 Table Raw demographic, histologic, and diagnostic data from a case-reference study (June 2010–December 2012) investigating the potential cause(s) of increased bottlenose dolphin (*Tursiops truncatus*) deaths in the northern Gulf of Mexico following the *Deepwater Horizon* oil spill. (XLSX)
